# First Phylogenetic Analysis of Malian SARS-CoV-2 Sequences Provides Molecular Insights into the Genomic Diversity of the Sahel Region

**DOI:** 10.3390/v12111251

**Published:** 2020-11-02

**Authors:** Bourema Kouriba, Angela Dürr, Alexandra Rehn, Abdoul Karim Sangaré, Brehima Y. Traoré, Malena S. Bestehorn-Willmann, Judicael Ouedraogo, Asli Heitzer, Elisabeth Sogodogo, Abderrhamane Maiga, Mathias C. Walter, Fee Zimmermann, Roman Wölfel, Markus H. Antwerpen

**Affiliations:** 1 Centre d’Infectiologie Charles Mérieux du Mali, BPE2283 Bamako, Mali; bourema.kouriba@cicm-mali.org (B.K.); abdoul.sangare@cicm-mali.org (A.K.S.); brehima.traore@cicm-mali.org (B.Y.T.); judicael.ouedraogo@cicm-mali.org (J.O.); elisabeth.sogodogo@cicm-mali.org (E.S.); abderrhamane.maiga@cicm-mali.org (A.M.); 2 Bundeswehr Institute of Microbiology, D-80937 Munich, Germany; angeladuerr@bundeswehr.org (A.D.); alexandrarehn@bundeswehr.org (A.R.); malenabestehornwillmann@bundeswehr.org (M.S.B.-W.); AsliHeitzer@bundeswehr.org (A.H.); mathias1walter@bundeswehr.org (M.C.W.); Feezimmermann@bundeswehr.org (F.Z.); romanwoelfel@bundeswehr.org (R.W.)

**Keywords:** SARS-CoV-2, phylogenetic analysis, Mali

## Abstract

We are currently facing a pandemic of COVID-19, caused by a spillover from an animal-originating coronavirus to humans occurring in the Wuhan region of China in December 2019. From China, the virus has spread to 188 countries and regions worldwide, reaching the Sahel region on 2 March 2020. Since whole genome sequencing (WGS) data is very crucial to understand the spreading dynamics of the ongoing pandemic, but only limited sequencing data is available from the Sahel region to date, we have focused our efforts on generating the first Malian sequencing data available. Screening 217 Malian patient samples for the presence of SARS-CoV-2 resulted in 38 positive isolates, from which 21 whole genome sequences were generated. Our analysis shows that both the early A (19B) and the later observed B (20A/C) clade are present in Mali, indicating multiple and independent introductions of SARS-CoV-2 to the Sahel region.

## 1. Introduction

SARS-CoV-2 (previously called 2019-nCoV) is a novel member in the genus of *Betacoronaviridae* and is responsible for the current, rapidly escalating COVID-19 pandemic. To this day, this virus, which has a lower pathogenicity than SARS-CoV but higher human-to-human transmissibility [1], caused more than 44 million infections and 1.1 million associated deaths worldwide (current as of 29 October 2020) [2].

The outbreak of the virus began in the city of Wuhan in China in December 2019, followed by the shift of the epicenter to Europe in mid-March 2020 [3]. From Europe and Asia, the wave of infections moved to North and South America, as well as Africa. The first COVID-19 case in the Sahel region was reported from Senegal on 2 March 2020 [4]. Within 3 weeks, the virus spread to all Sahel countries, including Burkina Faso, Mauritania, Chad, Niger, Sudan, and Eritrea, reaching Mali on the 25 March 2020 [5,6]. Since the Sahel region is currently facing several challenges, including a severe food and security crisis, it is difficult to judge the effects the COVID-19 pandemic will have on the already overwhelmed healthcare systems [7]. Especially in conflict-affected areas, where the population has only limited access to clean drinking water and handwashing facilities, hundreds of health centers are closed or not operating due to the poor security situation. Furthermore, social distancing measures are difficult to implement in dense African urban settings, and the national governments struggle to divide already insufficient budgets between health, food and security emergencies [8]. Therefore, it is not surprising that the medical and technical equipment, as well as the laboratory infrastructure in those countries, are sometimes outdated and not on a comparable level to most European countries or the United States. Hence, only limited genomic data of SARS-CoV-2 exists from the Sahel region, with no whole genome sequences being available from Mali to this date.

On 25 March 2020, the first two COVID-19 cases (a 49-year-old woman living in Bamako and a 62-year-old patient from Kayes, both returning from France) were confirmed in Mali [6]. To support the Malian public health system, SARS-CoV-2 diagnostic capabilities were established at the Centre d’Infectiologie Charles Mérieux du Mali (CICM-Mali) by training scientific personnel and by providing testing reagents, supported by the German Enhance and Enable Initiative’s Security Cooperation against Biological Threats in the G5 Sahel Region. Based on the given training, the CICM-Mali was prepared as the second and central diagnostic center to support the diagnosis of SARS-CoV-2 for Bamako and its surrounding regions and started its activities on 3 April 2020.

Since the confirmation of the first two cases in March 2020, a steady increase in the number of total cases, and the rapid spread of the SARS-CoV-2 virus within Mali, was observed. As of 26 October 2020, 3499 cumulative COVID-19 cases and 133 related deaths (a case fatality ratio of 3.8%) have been reported in Mali [9]. Based on the current data available, 13 out of 100,000 Malians tested positive, indicating a probably moderate risk for the Malian population to acquire COVID-19, especially considering the fact that mitigation strategies like social distancing cannot be applied to the same extent as in Europe [10], in addition to limited testing. In order to analyze the origins of existing infections and their distribution patterns, hence limiting the spread of SARS-CoV-2, sequencing data of COVID-19 positive cases are needed.

In this study, we analyzed the first Malian genome sequences of SARS CoV-2, originating from patients from Bamako and its surrounding villages. Using comparative genomics, we set the results in the context of available African genome sequences, thereby providing data of underrepresented regions, like the Sahel region, to the scientific community.

## 2. Materials and Methods

### 2.1. Patient Sampling

Nasopharyngeal or oropharyngeal samples were collected from 217 suspected patients using FLOQ swabs (Copan Diagnostics, Murrieta, CA, USA). Afterward, the swabs were stored in a UTM transport medium (Copan Diagnostics, Murrieta, CA, USA). Sampling was performed at the reference health centers of the affected health districts of Bamako, the capital city of Mali and the regions of Kayes, Koulikoro, Segou and Mopti. Samples were first collected at the National Institute of Public Health, then transferred to the Centre d’Infectiologie Charles Mérieux du Mali for analysis.

### 2.2. RNA Extraction and SARS-CoV-2 Detection

Viral RNA was extracted using the QIAamp Viral RNA Mini Kit (Qiagen, Hilden, Germany) and analyzed for the presence of SARS-CoV-2 at the Centre d’Infectiologie Charles Mérieux du Mali by reverse transcription quantitative PCR (RT-qPCR), according to the published protocol of [11], targeting the RdRp and E gene regions. A SuperScript III Platinum Polymerase qRT-PCR kit (ThermoFisher Scientific, Germering, Germany) was used for amplification. MS2 phages were added as an internal control. In order to allow simultaneous detection of the E and MS2 genes, the E singleplex assay of Corman et al. was converted into a multiplex assay by the addition of MS2-specific primers and probes (Cy5-labeled) [12]. Positive results obtained in the E gene assay were confirmed using the discriminatory RT-qPCR RdRp gene assay. The 38 samples, which tested positive for both the E and the RdRp genes, were sent to the Bundeswehr Institute of Microbiology (IMB) for whole genome sequencing.

### 2.3. Library Preparation and Sequencing

All samples were processed according to the published nCoV-2019 ARTIC sequencing protocol [13,14]. To this end, cDNA synthesis of the extracted RNA was performed according to the manufacturer’s instructions, using the SuperScript IV First-Strand Synthesis System (Invitrogen, Thermo Fisher Scientific, Dreieich, Germany). The subsequent multiplex PCRs (even and odd mixes) were performed using the V3 Primer Set in combination with the NEBNext Ultra II Q5 Master Mix (New England Biolabs, Frankfurt am Main, Germany). The only deviation from the original protocol was the reduction of the elongation time of the PCR to 45 s. The even and odd mixes of the PCR products were purified separately, and their DNA concentrations were determined using the Qubit™ dsDNA HS Assaykit (Thermo Fisher Scientific, Dreieich, Germany). The subsequent library preparation was executed with 25 ng of the even and odd PCR products. Finally, nanopore sequencing was performed using SQK-LSK109 chemistry on a 9.4.1 SpotON Flow Cell on the GridION system (Oxford Nanopore Technologies, Oxford, UK).

After passing sequence quality control, demultiplexing, which requires barcodes at both ends of the reads, was achieved using Porechop [15], and adapter trimming was achieved by the ARTIC pipeline [13] setting Wuhan-Hu-1 as the reference strain for read mapping.

When the ARTIC pipeline failed, or if the obtained consensus sequences were of too low a quality or incomplete, the extracted RNA was again converted to cDNA using the SuperScript IV First-Strand Synthesis System (Invitrogen, Thermo Fisher Scientific, Dreieich, Germany). After performing the second strand synthesis (NEBNext Ultra II Non-Directional RNA Second Strand Synthesis Module, New England Biolabs, Frankfurt am Main, Germany), an Illumina library was generated using the NEBNext Ultra II FS DNA Library Prep Kit. Additionally, a target enrichment step was incorporated prior to sequencing on an Illumina MiSeq (Illumina Inc., Berlin, Germany). For this purpose, specific baits of SARS-CoV-2 (myBaits Expert SARS-CoV-2, Arbor Sciences, BioCat, Heidelberg, Germany) were used according to the manufacturers’ instructions, and captured libraries were sequenced using Sequencing V2 Reagent chemistry at 300 cycles on a Micro Flow Cell (Illumina Inc., Berlin, Germany).

### 2.4. Phylogenetic Analysis

For the enrichment pipeline, Illumina reads were mapped to the Wuhan/Hu-1/2019 reference strain (GenBank accession MN908947) using bwa v0.7.17 [16]. The obtained bam files were added as a separate read group to the BAM files generated by the ARTIC pipeline using SAMtools [17]. Follow-up steps of the ARTIC pipeline were performed manually, using the combined BAM files as input. Additionally, variants were called for the Illumina mapping, using BCFtools v1.9-168 of the SAMtools package [17], and merged with the variant VCF files from the ARTIC pipeline, if necessary. All variations were screened against problematic_sites_sarsCov2.vcf for homoplastic sites or sequencing issues, which have the potential to adversely affect phylogenetic and evolutionary inference [18]. Final genomes were submitted to GISAID to make them publicly available.

SNP and phylogenetic analysis were performed using a local installation of the nextstrain.org pipeline [5]. For this, strains listed in GISAID and belonging to the African subset (*N* = 1203 current as of 22 June 2020) were included in the initial analysis and further filtered to minimize redundancy and selected by relevance to possible travel and/or trade routes (*N* = 73) for increasing readability while providing a broad phylogenetic overview at the same time.

### 2.5. Ethical Statement

The use of residual clinical specimens in this study was in accordance with the statement of the Central Ethics Committee of the German Medical Association on the further use of human body materials for the purposes of medical research of 20 February 2003. In addition, this study was approved by the Comite d’Ethique des Faculte de Medicine/d’Odontostomatologie et de Pharmacie (FMOS/FAPH) of the Universite des Science, des Techiques et de Technologies de Bamako (No. 2020/201/CE/FMOS/FAPH, 17 September 2020).

## 3. Results

### 3.1. Testing of Malian Patient Samples

**Figure 1:**
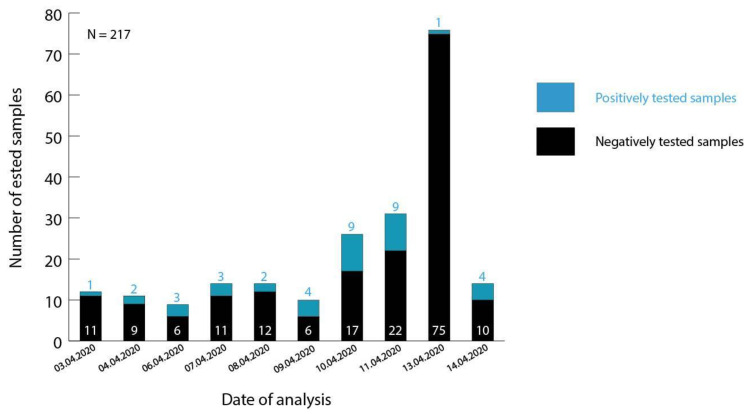
Number of positively confirmed samples analyzed at the Centre d’Infectiologie Charles Mérieux du Mali between 3 April 2020 and 14 April 2020. Patient samples tested on April 13 represent screened passengers of a return flight from Tunisia. Residual samples reflect suspected cases from Bamako and its surrounding regions. Samples containing the E and RdRp genes were confirmed as positive.

Between 3 April 2020 and 14 April 2020, 217 suspected cases (141 individuals living in Bamako and its surroundings and 76 passengers arriving from a return flight from Tunisia) were analyzed at the Centre d’Infectiologie Charles Mérieux du Mali for the presence of SARS-CoV-2 (Figure 1). A sample was only confirmed positive for a SARS-CoV-2 infection if the extracted RNA comprised of both the E gene and the RdRp genes. Via this approach, 38 patients (37/141 and 1/76) were diagnosed with COVID-19, representing 32.2% of all registered infections in Mali (*N* = 118) during the given course of time. The age of the infected patients ranged from 2–82 years, while significantly more male (23/38) than female (12/38) individuals tested positive for SARS-CoV-2.

The extracted RNA of positively tested patients was transferred to the Bundeswehr Institute of Microbiology for whole genome sequencing analysis, as qualified sequencing capacities were not available locally and this method is used as an extension of the currently used diagnostic algorithms.

### 3.2. Combination of Short-Read and Long-Read Sequencing Technologies, Resulting in the First 21 Full-Length Genomes of Mali

**Figure 2:**
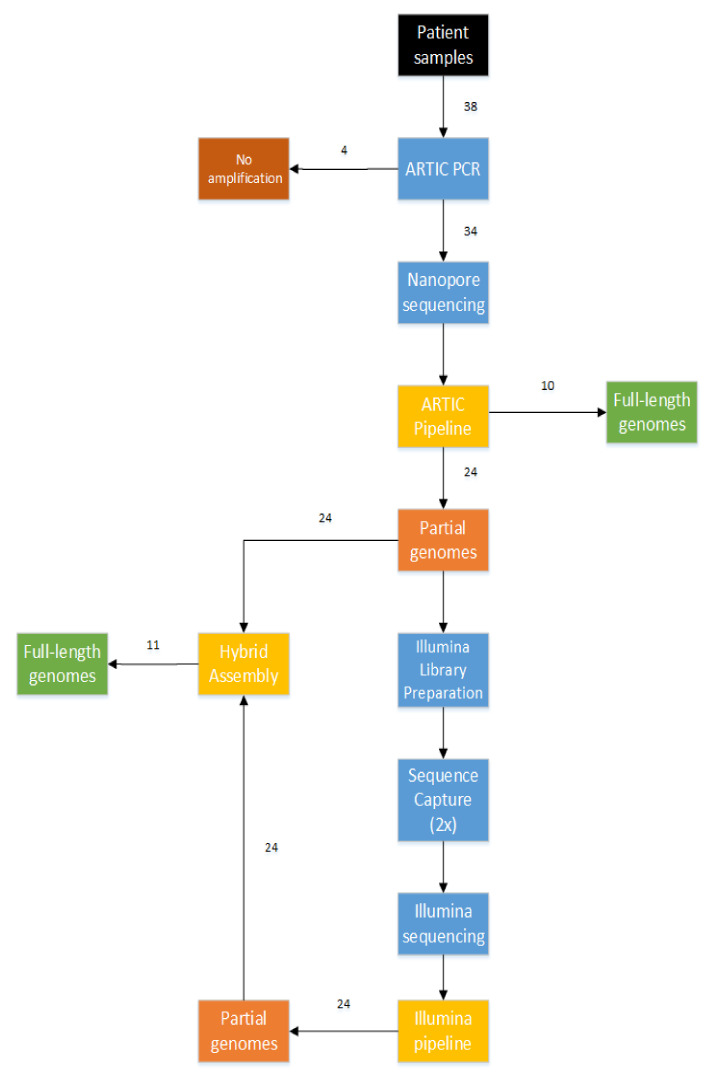
Workflow used to successfully sequence 21 out of 38 patient samples.

Ten full-length genome sequences could be successfully gained by using solely the ARTIC protocol, and a further 11 SARS-CoV-2 genomes could be obtained using a hybrid approach, combining the amplicons of the ARTIC protocol with the sequence reads from the enrichment workflow (see Figure 2 and Table S1). All genomes covered 29,749–29,858 nucleotides and reached a minimum sequencing depth of 40, thereby classifying themselves as high-quality genomes. Unfortunately, for 13 samples, even this advanced sequencing workflow resulted in only partially assembled genomes. Those isolates were neglected in further analysis. It is worth noticing that a clear correlation between the Ct values of the RdRp gene and successful sequencing was observed, showing that full-length genomes couldn’t be extracted from patient samples with Ct values higher than 32 (see Table S2).

Within the 21 full-length genomes, we noticed that, depending on the sequencing approach, some nucleotides of the 5′UTR and 3′UTR regions were missing. This phenomenon was especially observed in sequences generated only by reads based on the ARTIC protocol. However, in genomes assembled from reads of the hybrid approach (combination of the ARTIC protocol with Illumina sequencing), the 5′ UTR and 3′UTR regions were almost fully sequenced. Due to this fact, if only the ARTIC protocol was used, some de facto present SNPs in the 5′-UTR and 3′-UTR regions might not have been detected.

### 3.3. Testing of Malian Patient Samples, Where Malian SARS-CoV-2 Genomes Can Be Assigned to Two Different Lineages

**Figure 3:**
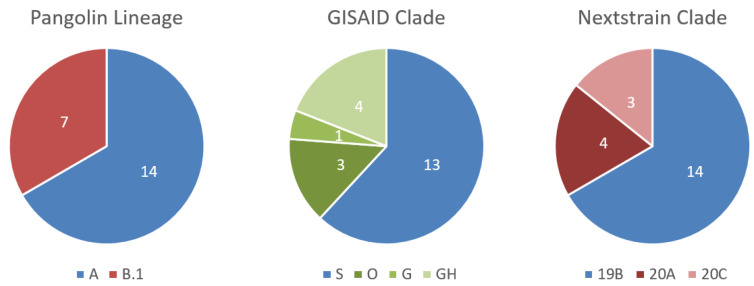
Sample assignment according to different nomenclatures used for SARS-CoV-2-classification.

Mapping of all Malian full-length genomes against the reference genome (Wuhan/Hu-1/2020) revealed SNPs in all sequences. Most of these were already known from other strains sequenced during this pandemic. Nineteen of the identified SNPs were synonymous, while 38 SNPs were non-synonymous and thus listed in Table 1 and Table S1, in addition to those located in the UTR regions. According to the quantity and position of the SNPs, the Malian SARS-CoV-2 genomes were assigned to clades. At the moment, three diverging nomenclatures exist: the Pangolin lineages based on the phylogenetic assignment of named global outbreak lineages (Pangolin) algorithm [19], the GISAID clades using the actual letters of the marker mutations [20] and the Nextstrain clades, which are defined by year and a letter code [21]. As the Pangolin lineages and the Nextstrain clades resulted in fairly similar classifications for the Malian SARS-CoV-2 genomes (see Figure 3), and it was unsure up to now which nomenclature would endure, we will discuss our results for both systems.

Strains sequenced in this study could be assigned to the two major clades that occurred so far during this pandemic, namely A and B.1, according to the Pangolin lineage. While 14 genomes could be assigned to lineage A, which represents the lineage of the first observed strain of the pandemic in Wuhan (Wuhan/Hu-01/2020), 7 genomes belonged to the currently worldwide and fast-spreading lineage B.1. According to the Nextstrain nomenclature, 14 strains belonged to the 19B cluster, while the remaining 7 strains were split into the 20A (4) and 20C (3) clades. One of the main differences between the A and B.1 lineages, and also the 19B and the 20* clades, was the presence of the mutation D614G in the spike protein, which became dominant with the shift of the pandemic from China to Europe in February 2020. The further partition of the 20A cluster into the 20C clade included the gain of two further amino acid substitutions, namely ORF1a:T265I and ORF3a:Q57H (see also Table 1).

**Figure 4:**
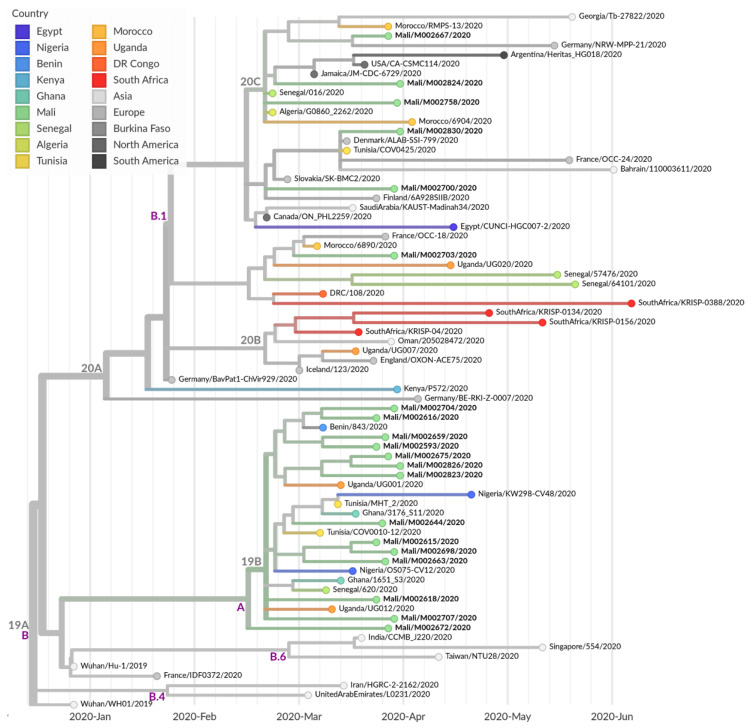
Maximum likelihood cladogram based on 47 representative genomes of African samples as, well as 26 genetic, closely related samples from Asia, Europe and North and South America. Nextstrain.org clade nomenclature is shown in gray and above branch nodes. Pangolin clade nomenclature is shown in violet and below branch nodes.

Interestingly, all 14 genomes of lineage A (19B) clustered very closely to each other and are currently dominating this clade in number (Figure 4). Based on the reported sampling dates of the corresponding genome sequences, this clade was originally dominated by Asian samples (country confidence: Asia 81%, Europe 10%, Mali 4% and Oceania 2%), while recently African, and especially Malian, genomes became more apparent (country confidence: Asia 58%, Europe 5%, Mali 36% and Oceania 0%).

In contrast, the remaining seven genomes were widely dispersed within the B.1 lineage (20A/C) (see Figure 4), such as the genome sequence isolated from the positively tested Malian passenger (M00258), who returned from Tunisia. It also belonged to clade B.1, which might assume a possible infection outside of Mali. However, as no information about symptom onset or Tunisia entry date was available from this passenger, it could not be ruled out that the COVID-19 infection was already acquired in Mali prior departure to Tunisia. Furthermore, nearest neighbor sequences to Malian genomes of this lineage were also locally widespread and originated from all over the world.

### 3.4. Further Characterization of SARS-CoV-2 Genomes Derived from Malian Patients

Besides the division of the sequenced full-length genomes into A (19B) and B (20A/C) clusters, two additional observations are worth mentioning. The first one concerns sample M002672, belonging to the A (19B) lineage. Here, a 9 bp deletion at the nucleotide position 685, resulting in a 3 amino-acid deletion of the protein ORF1a, was detected. This microdeletion was already described in SARS-CoV-2 genomes derived from countries in the northern hemisphere, like Iceland, Sweden, England, Wales, Canada and the United States [22], but had, to our knowledge, not been detected in African samples to that point. Unfortunately, a phenotypic function of this microdeletion remains elusive to this day.

The second observation was made in samples M002673 and M002707 at positions 14,408 and 18,973, respectively. In both samples, quasispecies, indicated by the simultaneous occurrence of at least two isolates, were detected at the described position. Table 1 and Table S1 lists only the dominant and hence more frequently occurring mutation. As the quasispecies were observed independent of the applied sequencing technology (Nanopore and Illumina), artificially introduced mutations via, for example, PCR can be excluded. The C14408T mutation, which leads to the P314L conversion in ORF1b, is a known mutation and, next to D614D, another hallmark for the B (20*) cluster. This mutation seems to provoke a positive effect for the virus, as it is stable when present in this lineage. In contrast, the G18973T mutation, leading to the amino acid exchange V1836F, was only detected once before in a German cluster (the example strain Germany/NRW-MPP-24/2020), but did not obviously manifest as reliable phylogenetic marker. In our sample, this allelic variant was observed with a frequency of one-third at a read depth of 235. Nevertheless, we had the rare opportunity to witness the evolution of the virus within a human sample.

Regarding the variant screening, we found only one variant in two strains, M002593 and M002659, which may confound evolutionary interpretations in G11083T. Position 11,083 is a major homoplastic site in Orf1ab [23] which is geographically ubiquitous, and it appeared in strain M002659 both in the Illumina and MinION reads.

## 4. Discussion

Up until now, only limited sequencing data of Northern Africa and the Sahel region was available, compared with other regions of the world like America, Asia or Europe. Viral genomes spreading on the African continent are mainly accessible from states like South Africa [24], the Democratic Republic of Congo (DRC) and Kenya [25]. For the Sahel region, genomic information of SARS-CoV-2 is only provided by Nigeria [26] and Senegal [5,27], while data from countries like Mauritania, Niger, Burkina Faso, Chad, Eritrea and Sudan are still missing. With our full-length SARS-CoV-2 genomes originating from Mali, we want to contribute to the cohort of sequencing data from the Sahel region, thereby enhancing the knowledge of the genomic diversity of COVID-19 infections in this country and helping the reconstruction of the geographic spread.

The Sahel region separates the northern African states (Morocco, Algeria, Tunisia, Libya and Egypt), which are historically (and up to today) linked to Europe, from the indigenous and distinct cultures from Central Africa. This unique geographical position makes the Sahel region exceptionally interesting for studying the introduction to North Africa of the COVID-19 pandemic. Our results show that two SARS-CoV-2 lineages, namely A and B.1, are circulating in Mali, with lineage A being causative for about two-thirds of the infections. Even though our findings might be biased, because mainly individuals from Bamako and its surroundings were sampled, the presence of the two lineages strongly suggests at least two different and independent introduction points of the SARS-CoV-2 infection in Mali.

Since the A lineage contains the initial emerging pandemic strain, as well as Asian genomes [19], we suggest a very early, but probably unrecognized, onset of infections in Mali. The reasons that most likely encouraged the non-detection of the virus are, on the one hand, the fragile and overstretched health care system, and on the other hand, the transmission through patients with only mild symptoms being misdiagnosed, or even not recognized at all. These listed reasons might also explain the relatively high mortality rate (5%) and the high estimated number of unreported cases in Mali.

In contrast, the B.1 lineage is comprised of sequences associated with the large Italian outbreak in February 2020 and represents, to date (current as of date 21 July 2020), together with its sublineage B.1.1, the worldwide prevalent virus clade [19]. The African continent, especially South Africa, is dominated by lineages B (its origin still lying in Asia) and B.1 [24]. In Mali, only one-third of the patient samples were assigned to lineage B.1, thus giving rise to speculation that this viral lineage was imported at a later point in time. As the nearest neighbors of our Malian genomes belong to the B.1 lineage and are geographically spread out (Europe, United States, Canada and Northern Africa), we can exclude a single event as the origin of introduction of the B.1 lineage into Mali. More likely, multiple travel-associated transfers from various countries have caused the spread of the B.1 lineage within Mali. Unfortunately, restrictive measures in order to contain the circulation within Mali are only possible to a limited extent, since social distancing measures endanger vital income for survival and are hence not fully feasible.

To follow the three main rules of outbreak management, namely test, trace and isolate, the German Enhance and Enable Initiative’s Security Cooperation against Biological Threats in the G5 Sahel Region has established SARS-CoV-2 diagnostics at the Centre d’Infectiologie Charles Mérieux du Mali in Bamako, seeing the importance of strengthening and increasing testing capacities in Mali and Sahelian G5 partner countries. In doing so, we believe that a prompt identification of the source of infection will not only lead to a backtracking and isolation of individuals or groups, but also to reasonable national measures to restrict or terminate the SARS-CoV-2 spread within the country and hence worldwide.

## Figures and Tables

**Table 1:** Overview of observed mutations, which occurred in at least two patients. Het = heterozygous, hp = highly homoplastic, syn = synonymous. For full details, please refer to Table S1.

Region	Mutation (nt)	Mutation (aa)	Counts	Samples
5′UTR	C241T	-	6	M002667,M002672,M002703,M002758,M002824,M002830
ORF1a	A361G	syn	2	M002644
	C1059T	T265I	3	M002667,M002758,M002824
	C1968T	T563I	2	M002672,M002703
	C2416T	syn	2	M002700,M002830
	C3037T	syn	7	M002667,M002673,M002700,M002703,M002758,M002824,M002830
	C8782T	syn	14	M002593,M002615,M002616,M002618,M002644,M002659,M002663,M002672,M002675,M002698,M002704,M002707,M002823,M002826
	G11083T(hp)	L3606F	2	M002593,M002659
	G11417T	V3718F	3	M002675,M002823,M002826
	C11747T	syn	2	M002672,M002703
ORF1b	C14408T	P314L	7	M002667,M002673,M002700,M002703,M002758,M002824,M002830
	C15324T	syn	2	M002673,M002703
	C16658T(het)	T1064I	2	M002675,M002707
S	G22468T	syn	4	M002615,M002644,M002663,M002698
	A23403G	D614G	6	M002667,M002700,M002703,M002758,M002824,M002830
ORF3a	G25563T	Q57H	5	M002667,M002700,M002758,M002824,M002830
	C25904T	S171L	3	M002615,M002663,M002698
ORF8	T28144C	L84S	13	M002593,M002615,M002616,M002618,M002644,M002659,M002663,M002675,M002698,M002704,M002707,M002823,M002826
N	G28878A	S202N	14	M002593,M002615,M002616,M002618,M002644,M002659,M002663,M002672,M002675,M002698,M002704,M002707,M002823,M002826
3′UTR	G29742A	-	12	M002593,M002615,M002616,M002618,M002644,M002659,M002663,M002672,M002675,M002704,M002707,M002826

## Supplementary Materials

The following are available online at https://www.mdpi.com/1999-4915/12/11/1251/s1, Table S1: Ct values of diagnostic PCRs and outcome of sequencing. Samples sequenced successfully are highlighted yellow, Table S2: Overview of patient information, sequencing results and used technology results of Malian samples.
