# Supplementary material for: First Phylogenetic Analysis of Malian SARS-CoV-2 Sequences Provides Molecular Insights into the Genomic Diversity of the Sahel Region
Source: Viruses. 2020 Nov 2;12(11):1251. doi: 10.3390/v12111251 (PMC7692263; doi:10.3390/v12111251)
Supplement: Supplementary file 1 [file viruses-12-01251-s001.pdf]

First phylogenetic analysis of Malian SARS-CoV-2 sequences provides molecular insights into the genomic diversity of the Sahel region

Table 1. Ct values of diagnostic PCRs and outcome of sequencing. Samples sequenced successfully are highlighted yellow.

ID	Ct E gene	Ct RdRp gene	sequencing succesfull
M002593	27,48	29,43	y
M002615	28,51	32,83	y
M002616	35,14	32,71	y
M002618	30,2	32,21	y
M002644	26,6	27,62	y
M002659	28,03	30,27	y
M002663	34,08	34,2	y
M002667	28,1	29,08	y
M002672	22,96	24,37	y
M002673	21,23	21,93	y
M002675	29,55	30,06	y
M002698	30,19	32,61	y
M002700	30,02	31,85	y
M002703	26,07	28,37	y
M002704	27,13	30,16	y
M002707	16,5	19,05	y
M002758	19,44	25,11	y
M002823	24,37	27,27	y
M002824	29,07	31,62	y
M002826	30,21	32,07	y
M002830	21,42	24,43	y
M002609	38,61	37,01	n
M002610	40,24	37,58	n
M002627	35	38,98	n
M002634	34,44	35,9	n
M002635	34,38	36,09	n
M002638	34,36	34,07	n
M002652	34,35	36,23	n
M002656	34,57	37,15	n
M002658	32,01	33,94	n
M002662	36,66	37,01	n
M002664	34,44	33,97	n
M002676	35,42	35,14	n
M002680	34,82	35,13	n
M002688	29,4	33,86	n

M002708	35,08	36,67	n
M002712	34,05	36,36	n
M002714	31,92	34,3	n

Table 2. Overview of patient information, sequencing results and used technology results of Malian samples (M = Male, F = Female, n.d. = not determined, A = ARTIC Protocol, H = Hybrid assembly of ARTIC protocol and bait-based enrichment using Illumina MiSeq, het = heterozygous, hp = highly homoplasic). Only non-synonymous mutations and deletions are listed.

Sample ID	Patient Sex, Age	Location	AccNo GISAID	Lineage (Nextstrain Clade)	Seq-Methods	Reference covered positions	SNPs (AA exchanges)
M002593	M, 50-60	Bamako	EPI_ISL487446	A (19B)	A	55 - 29835	G11083T (ORF1a: L3606F, hp)<br>T28144C (ORF8:L84S)<br>G28878A (N:S202N)<br>C29585T (ORF10:P10S)<br>G29742A (3'UTR)
M002615	M, 40-50	Sévaré	EPI_ISL_4874	A (19B)	H	55 - 29863	C25904T (ORF3a:S171L)<br>T28144C (ORF8:L84S)<br>G28878A (N:S202N)<br>G29742A (3'UTR)
M002616	F, 40-50	Bamako	EPI_ISL_487448	A (19B)	H	19 - 29776	C14189T (ORF1b:A241V)<br>T28144C (ORF8:L84S)<br>G28878A (N:S202N)<br>G29742A (3'UTR)
M002618	M, 60-70	Bamako	EPI_ISL_487449	A (19B)	H	16 - 29871	G581A (ORF1a:V106I)<br>A10323G(ORF1a:K3353R)<br>T28144C (ORF8:L84S)<br>G28878A (N:S202N)<br>G29742A (3'UTR)
M002644	F, 50-60	Bamako	EPI_ISL_487450	A (19B)	A	55 - 29835	A14255G (ORF1b:K263R)<br>T28144C (ORF8:L84S)<br>G28878A (N:S202N)<br>G29742A (3'UTR)
M002659	M, 20-30	Bamako	EPI_ISL_487451	A (19B)	H	16 - 29872	G11083T (ORF1a:L3606F, hp)<br>C26366T (E:A41V)<br>T28144C (ORF8:L84S)<br>G28878A (N:S202N)<br>G29742A (3'UTR)
M002663	M, 20-30.	Bamako	EPI_ISL_487452	A (19B)	H	16 - 29860	C10626T (ORF1a:A3454V)<br>C25904T (ORF3a:S171L)<br>C25936T (ORF3a:H182Y)<br>T28144C (ORF8:L84S)<br>G28878A (N:S202N)<br>G29742A (3'UTR)

M002667	M, 20-30	Bamako	EPI_ISL_487453	B.1 (20C)	A	55 - 29835	C241T(5′ UTR)<br>C1059T (ORF1a:T265I)<br>C14408T (ORF1b:P314L)<br>A23403G (S:D614G)<br>G25563T (ORF3a:Q57H)
M002672	F, 50-60	Bamako	EPI_ISL_487454	A (19B)	A	55 - 29826	C241T(5′ UTR)<br>685-693 gap (9 nt)<br>C1968T (ORF1a:T563I)<br>G28878A (N:S202N)
M002673	F, 80-90	Bamako	EPI_ISL_487455	B.1 (20A)	A	55 - 29835	C14408T (ORF1b:P314L, het.)
M002675	M, 60-70	Bamako	EPI_ISL_487456	A (19B)	H	25 - 29859	G11417T (ORF1a:V3718F)<br>C16658T (ORF1b:T1064I het.)<br>T28144C (ORF8:L84S)<br>G28878A (N:S202N)<br>G29742A (3′UTR)
M002698	F, 60-70	Bamako	EPI_ISL_487457	A (19B)	H	19 - 29800	C25904T (ORF3a:S171L)<br>T28144C (ORF8:L84S)<br>G28878A (N:S202N)
M002700	M, 60-70	Bamako	EPI_ISL_487458	B.1 (20A)	H	126 - 29873	G11128A (ORF1a:M3621I)<br>C14408T (ORF1b:P314L)<br>A23403G (S:D614G)<br>C25433T (ORF3a:T14I)<br>G25563T (ORF3a:Q57H)<br>T26038C (ORF3a:S216P)<br>29554-29602 gap (48 nt)
M002703	F, 80-90.	Bamako	EPI_ISL_487459	B.1 (20A)	H	16 - 29839	C241T(5′ UTR)<br>C1968T (ORF1a:T568I)<br>C14408T (ORF1b:P314L)<br>G15743A (ORF1b:S759N)<br>C22290T (S:A243V)<br>A23403G (S:D614G)
M002704	M, 50-60	Bamako	EPI_ISL_487460	A (19B)	H	17 - 29870	T28144C (ORF8:L84S)<br>G28878A (N:S202N)<br>G29742A (3′UTR)
M002707	M, 60-70	Bamako	EPI_ISL_487461	A (19B)	A	55 - 29835	C16658T (ORF1b:T1064I)<br>G18973T (ORF1b:V1836F, het.)<br>T28144C (ORF8:L84S)<br>G28878A (N:S202N)<br>G29742A (3′UTR)
M002758	M, 70-80	Bamako	EPI_ISL_487462	B.1 (20C)	A	55 - 29835	C241T(5′ UTR)<br>C1059T (ORF1a:T265I)<br>G10974A (ORF1a S3570N)<br>G12292T (ORF1a M4009I)<br>C14408T (ORF1b:P314L)<br>A23403G (S:D614G)<br>G25563T (ORF3a:Q57H)<br>C26198T (ORF3a:T269M)<br>G29745A (3′UTR)

M002823	M, 40-50	Bamako	EPI_ISL_487463	A (19B)	A	55 - 29835	C2189T (ORF1a:L642F)<br>G11417T (ORF1a:V3718F)<br>T28144C (ORF8:L84S)<br>G28878A (N:S202N)
M002824	M, 0-10	Bamako	EPI_ISL_487464	B.1 (20C)	A	55 - 29835	C241T(5′ UTR)<br>A963T (ORF1ab:E233V)<br>C1059T (ORF1a:T265I)<br>C11916T (ORF1a:S3884L)<br>C14408T (ORF1b:P314L)<br>A23403G (S:D614G)<br>G25563T (ORF3a:Q57H)
M002826	F, 30-40	Bamako	EPI_ISL_487465	A (19B)	H	19 - 29838	G11417T (ORF1a:V3718F)<br>T28144C (ORF8:L84S)<br>G28878A (N:S202N)<br>G29742A (3′UTR)
M002830	M, 80-90	Bamako	EPI_ISL_487466	B.1 (20A)	A	55 - 29835	C241T(5′ UTR)<br>G8371T (ORF1a:Q2702H)<br>C12465T (ORF1a:A4067V)<br>C14408T (ORF1b:P314L)<br>G20102T (ORF1b:S2212I)<br>A23403G (S:D614G)<br>G25563T (ORF3a:Q57H)
